# Combined Application of Dual-Wavelength Fluorescence Monitoring and Contactless Thermometry during Photodynamic Therapy of Basal Cell Skin Cancer

**DOI:** 10.17691/stm2020.12.3.06

**Published:** 2020-06-28

**Authors:** A.M. Mironycheva, M.Yu. Kirillin, A.V. Khilov, A.Sh. Malygina, D.A. Kurakina, V.N. Gutakovskaya, I.V. Turchin, N.Yu. Orlinskaya, I.L. Shlivko, I.A. Klemenova, O.E. Garanina, S.V. Gamayunov

**Affiliations:** Assistant, Department of Skin and Venereal Diseases; Privolzhsky Research Medical University, 10/1 Minin and Pozharsky Square, Nizhny Novgorod, 603005, Russia; Junior Researcher, Group for Skin Neoplasm Study, University Clinic; Privolzhsky Research Medical University, 10/1 Minin and Pozharsky Square, Nizhny Novgorod, 603005, Russia; Senior Researcher, Laboratory of Biophotonics; Federal Research Center Institute of Applied Physics, Russian Academy of Sciences, 46 Ulyanova St., Nizhny Novgorod, 603950, Russia;; Junior Researcher, Laboratory of Biophotonics; Federal Research Center Institute of Applied Physics, Russian Academy of Sciences, 46 Ulyanova St., Nizhny Novgorod, 603950, Russia;; Junior Researcher, Group for Skin Neoplasm Study, University Clinic; Privolzhsky Research Medical University, 10/1 Minin and Pozharsky Square, Nizhny Novgorod, 603005, Russia; Junior Researcher, Laboratory of Biophotonics; Federal Research Center Institute of Applied Physics, Russian Academy of Sciences, 46 Ulyanova St., Nizhny Novgorod, 603950, Russia;; Student; Privolzhsky Research Medical University, 10/1 Minin and Pozharsky Square, Nizhny Novgorod, 603005, Russia; Head of the Laboratory of Biophotonics; Federal Research Center Institute of Applied Physics, Russian Academy of Sciences, 46 Ulyanova St., Nizhny Novgorod, 603950, Russia;; Acting Head of the Department of Pathologic Anatomy; Privolzhsky Research Medical University, 10/1 Minin and Pozharsky Square, Nizhny Novgorod, 603005, Russia; Chief Researcher, University Clinic; Privolzhsky Research Medical University, 10/1 Minin and Pozharsky Square, Nizhny Novgorod, 603005, Russia; Chief Researcher, Group for Skin Neoplasm Study, University Clinic; Privolzhsky Research Medical University, 10/1 Minin and Pozharsky Square, Nizhny Novgorod, 603005, Russia; Head of the Department of Skin and Venereal Diseases; Privolzhsky Research Medical University, 10/1 Minin and Pozharsky Square, Nizhny Novgorod, 603005, Russia; First Vice-Rector, Professor, Department of Skin and Venereal Diseases; Privolzhsky Research Medical University, 10/1 Minin and Pozharsky Square, Nizhny Novgorod, 603005, Russia; Assistant, Department of Skin and Venereal Diseases; Privolzhsky Research Medical University, 10/1 Minin and Pozharsky Square, Nizhny Novgorod, 603005, Russia; Researcher, University Clinic; Privolzhsky Research Medical University, 10/1 Minin and Pozharsky Square, Nizhny Novgorod, 603005, Russia; Head Physician Nizhny Novgorod Regional Oncologic Dispensary, 11/1 Delovaya St., Nizhny Novgorod, 603163, Russia

**Keywords:** basal cell skin cancer, photodynamic therapy, fluorescence diagnosis, contactless thermometry.

## Abstract

**The aim of the study** was to assess the capabilities of combined application of dual-wavelength fluorescence visualization and contactless skin thermometry during photodynamic therapy monitoring (PDT) of basal cell cancer.

**Materials and Methods.:**

The study was performed at the University Clinic of Privolzhsky Research Medical University (Nizhny Novgorod). Nine clinically, dermatoscopically, and histologically verified foci of basal cell skin cancer were exposed to PDT sessions (wavelength of 662 nm, light dose density of 150 J/cm^2^) with systemic application of chlorin-based photosensitizer Fotoditazin. A semiconductor laser system Latus-T (Russia) was employed for irradiation. Dual-wavelength fluorescence visualization and contactless thermometry with an IR pyrometer were used to monitor the PDT sessions.

**Results.:**

The PDT sessions of nine foci of basal cell cancer were carried out under the control of fluorescence imaging and contactless thermometry. Photosensitizer photobleaching in all foci amounted to 40% signifying a percent of photosensitizer involved in the photodynamic reaction. It has been shown that the combined employment of dual-wavelength fluorescence monitoring and contactless thermometry during the PDT of basal cell skin cancer allows oncologists to control simultaneously the degree of photosensitizer photobleaching and the depth of the photodynamic effect in tissues, the extent of involving the mechanisms associated with hyperthermia as well as the correctness of the procedure conducting. In the course of 9-month dynamic follow-up after the treatment, no clinical and dermatoscopic signs of recurrence were found.

**Conclusion.:**

A bimodal control of PDT enables the assessment of the correctness and efficacy of the procedure performance. The contactless control of tissue heating allows ensuring the temperature mode for hyperthermia realization, while the fluorescence monitoring makes it possible to evaluate the accumulation of the photosensitizer in the tumor and the depth of the PDT action as well as to predict the procedure efficacy based on the photobleaching data. The complementary use of these techniques allows the adjustment of the mode directly in the course of the PDT procedure. The acquisition of the sufficient statistical data on the combined monitoring will result in the development of a novel PDT protocol.

## Introduction

In 2018, a total of 624,709 primary cases of malignant neoplasms were revealed in the Russian Federation, with the value increase by 1.2% as compared to 2017. Skin is one of the leading localizations in the general structure of the oncologic morbidity (12.6% among non-melanoma cancers, 14.4% among all cancers including melanoma). The number of patients with skin malignant neoplasms (excluding melanoma) per 100,000 population in Russia amounted to 233.4 in 2008 and 298.2 in 2018 [1].

Basal cell skin cancer (BCSC) is an epithelial malignant tumor developing in epidermis or hair follicles from basal keratinocytes with locally destructive growth and rare metastasizing. Clinical recommendations for treatment of non-melanoma skin cancers imply an individual approach in selecting the treatment tactics taking into consideration the localization, extension of the tumor process, prognostic factors, general condition of the patient including the severity of comorbid pathologies. Traditional methods of BCSC management include surgery and radiative (close-distance radiation therapy) approaches. If BCSC foci are located in hard-to-reach places, or in case of multiple foci, or a chronic somatic pathology in the medical history, treatment may appear to be complicated or impossible [2, 3].

Photodynamic therapy (PDT) is one of the organ-preserving techniques. Its application gives the opportunity to achieve maximal organ-preserving and functional results and adequate oncological efficacy [4]. PDT is based on the ability of photosensitizers to be accumulated selectively in tumor tissues. When exposed to laser radiation of a specified wavelength, a photochemical reaction takes place, which results in production of singlet oxygen and other active forms of oxygen leading to selective death of tumor cells. Local disturbance of microcirculation in the form of vasoconstriction, vascular thrombosis, and stasis is an additional mechanism of PDT disordering blood supply to the tumor and promoting its death [5].

One of the most common classes of photosensitizers actively used in Russia is the chlorin line of photosensitizers, which are characterized by two peaks in the absorption spectrum with the central wavelengths of 402 and 662 nm. An additional advantage of these photosensitizers consists in their fluorescence properties providing the possibility of using fluorescence visualization to monitor their accumulation and photobleaching [6]. Optical properties of the skin at 402 and 662 nm wavelengths differ significantly: skin absorption depending mainly on water and hemoglobin is much lower at 662 nm as compared to 402 nm [7]. In this connection, dual-wavelength fluorescence excitation gives additional information, since at 405 nm wavelength it provides a signal from the superficial tissue layers whereas at 660 nm the probing depth appears to be essentially greater [8, 9].

PDT is known to destroy cellular elements and preserve collagen structures which is a favorable background for tissue reparation, scar formation, good cosmetic results [10, 11].

An important aspect of PDT consists in the control of the temperature mode. Given a sufficient amount of statistical data, the temperature of tissue heating during the PDT procedure may be an additional prognostic factor: a significant deviation of the heating value from typical figures may serve as an indicator for correction of the treatment tactics. On the one hand, tumor heating can stimulate its growth, on the other hand, some complementary mechanisms causing destructive effect on the tumor may be triggered when the temperature reaches a certain level [12].

One of the directions of PDT development is to work out methods of intraoperative non-invasive control for predicting the results of the therapy and to create optimal treatment protocols.

**The aim of the study** was to assess the capabilities of a combination of dual-wavelength fluorescence imaging and contactless skin thermometry in the course of the control of photodynamic therapy of basal cell cancer in order to achieve radical recovery without undesirable side-effects.

## Materials and Methods

For PDT procedures, BCSC foci were selected in a 68-year-patient who was diagnosed to have “basal cell cancer of the trunk skin, cT_3_N_0_M_0_, stage II, multicentric growth”. Examination and PDT treatment assisted by bimodal monitoring were carried out at the Scientific and Practical Center for Diagnosing and Treatment of Skin Tumors of the University Clinic of Privolzhsky Research Medical University (Nizhny Novgorod, Russia). The study was performed in compliance with the Declaration of Helsinki (2013) and approved by the Ethical Committee of Privolzhsky Research Medical University. Written informed consent was obtained from the patient.

The patient was administered the PDT procedures after he had undergone multiple combined treatment. The disease was established to be progressing: new foci appeared on the skin of the trunk and shoulders. Hereditary syndromes, which might cause the emergence of multiple foci of basal cell cancer, were excluded.

**Figure 1:**
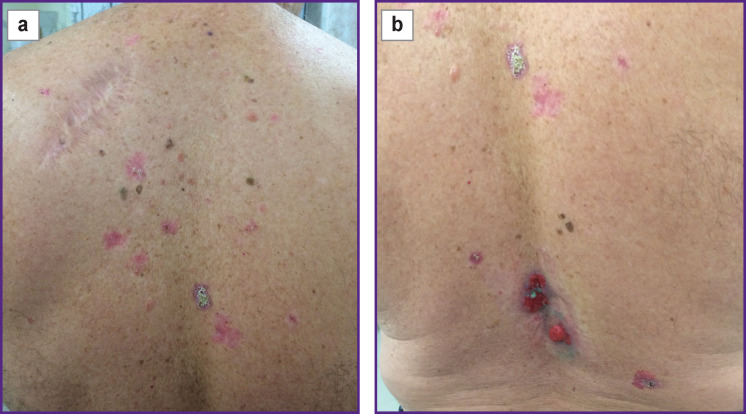
Multiple foci of basal cell skin cancer on the back (а) and lumber region (b)

Clinically, multiple neoplasms were revealed on the skin of the back and shoulders manifested by irregular-shaped spots with sharp boundaries and nodes of 3 cm in diameter, pink-colored, some foci were with serous and hemorrhagic crusts on the surface (Figure 1 (a)). On the skin of the lumbar region exophytic nodes of the pink-red color with a serous and hemorrhagic discharge were noted (Figure 1 (b)).

**Figure 2:**
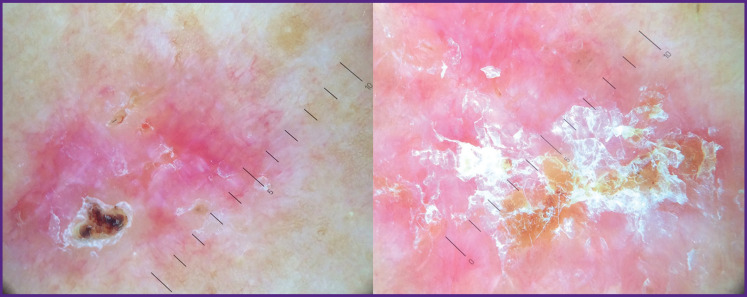
Dermoscopic picture of two superficial elements of basal cell skin cancer of the back

Dermatoscopic examination visualized structureless areas of the milk-white color, grey globules, white crystal structures, structureless areas of brown-yellow and white-yellow color, and polymorphic vessels (Figure 2).

**Figure 3:**
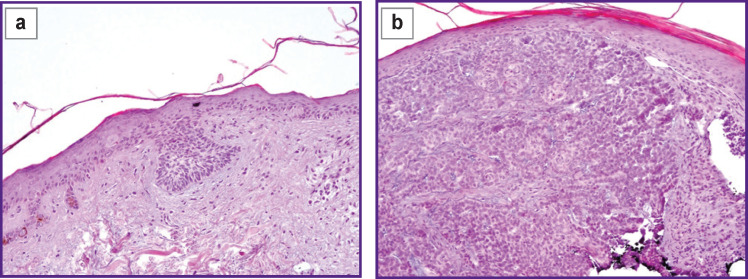
Pathomorphological picture of the basal cell skin cancer of the back: (а) superficially spreading basal cell carcinoma; (b) solid variant of basal cell carcinoma; stained with hematoxylin and eosin; ×100

Material from all foci was collected for histological investigation by a shave biopsy method, the findings of the investigation confirmed the diagnosis of BCSC (Figure 3).

Taking into account multiple BCSC foci, the previous treatment, and comorbid pathology, the patient was proposed a combined therapy: surgical treatment of the nodal BCSC foci and PDT of the foci of superficial BCSC form on the back.

A session of PDT was conducted with intravenous injection of photosensitizer Fotoditazin (registration No.FSR 2012/13043) with a total dose of 100 mg (calculated as 1 mg/kg of body mass). A semiconductor laser device Latus-T (Actus Ltd. Co., Russia; registration No.FSR 2010/09207) at the wavelength of 662±1 nm was used as a source of radiation. The power density of the device was 0.3 W/cm^2^, the dose density for each BCSC focus was 150 J/cm^2^.

Monitoring of the PDT procedure for BCSC treatment was performed using dual-wavelength fluorescence imaging system developed at the Federal Research Center Institute of Applied Physics of the Russian Academy of Sciences (Nizhny Novgorod, Russia) [8, 9]. Fluorescence monitoring is based on the registering photobleaching of the photosensitizer accumulated in the tumor manifested by the reduction of the fluorescence response signal level during the procedure. The value of more than 40% is considered a good indicator [13]. IR pyrometer (Optris, Germany) was used for a contactless temperature control.

## Results

### Dual-wavelength fluorescence monitoring.

**Figure 4:**
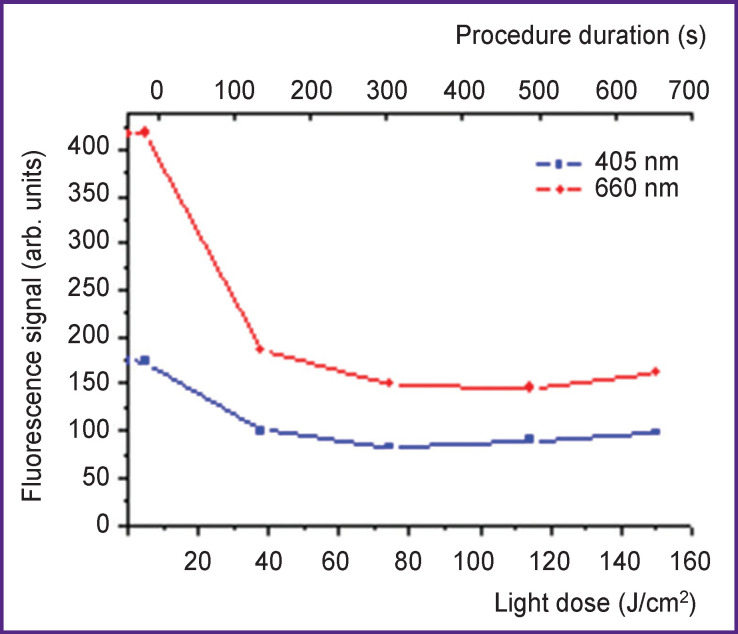
Dynamics of the fluorescence signal at 405 and 660 nm wavelength excitation in the process of photodynamic therapy of basal cell skin cancer

A typical dynamics of fluorescence signal during photodynamic therapy of BCSC at the excitation wavelengths of 405 and 660 nm is presented in Figure 4. Monotonic decrease of the signal is an indicator of photosensitizer bleaching, whereas a possible small increase of fluorescence level may signify the inflow of the photosensitizer to the treated place with blood circulation.

The photobleaching dynamics at λ=405 nm characterizes bleaching in the superficial layers (~200 μm). Since the photosensitizer is injected intravenously for BCSC treatment, the delivery to the foci occurs via the blood flow, and its major amount is accumulated at great depths, one may expect that the photobleaching value when probing at λ=660 nm (penetrates as deep as about 2 mm) will be greater than that at λ=405 nm, which was observed in the considered case.

**Figure 5:**
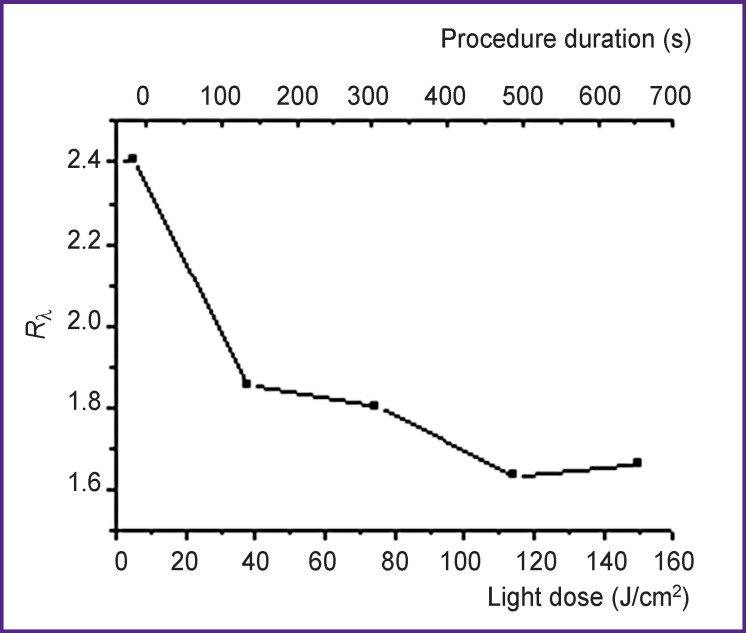
Dynamics of the fluorescence signals ratio *R*_λ_ at 405 and 660 nm wavelength excitation in the process of photodynamic therapy of basal cell skin cancer

This effect may be more evident when analyzing the dynamics of fluorescence signals ratio:


$$R_{\lambda }=\frac{I_{f}(\lambda =660\text{​ nm})}{I_{f}(\lambda =405\text{​ nm})},$$

where *I_f_* (λ) is the fluorescence signal at the excitation wavelength λ (Figure 5). Decrease of *R*_λ_ indicated a more intensive photobleaching at large depths compared to the superficial layers.

The results of numerical modeling [14] of the fluorescence monitoring of the systemic photosensitizer injection allowed us to estimate the depth of penetration equal to 0.6–0.8 mm based on the measured values of *R*_λ_.

It should be noted that photobleaching amounted to more than 40% for all procedures performed, which is a predictor of a successful outcome of the procedure [13].

### Non-invasive temperature monitoring.

The developed protocol of the PDT procedure for BCSC included also a non-invasive temperature monitoring with an IR pyrometer which enables the assessment of the area heating degree being an indirect indicator of the locally absorbed dose. The temperature measurement simultaneously with the PDT procedure makes it possible to control the achievement of the hyperthermia level, at which complementary mechanisms affecting the tumor may be triggered.

**Figure 6:**
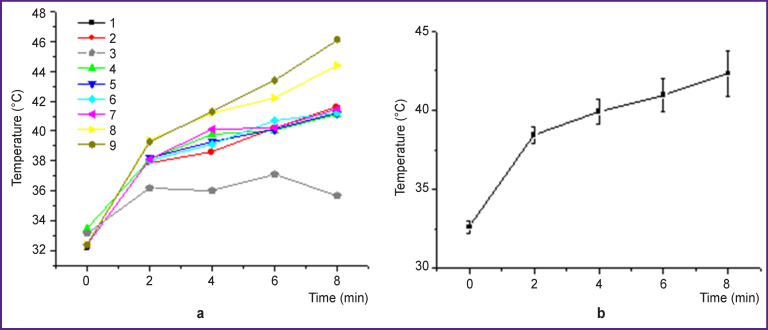
Temperature dynamics in the process of photodynamic therapy: (а) in each radiated foci (*1*–*9*); (b) averaged across all foci

The results of the temperature monitoring (Figure 6) show that the temperature of the treated area exceeded 41°C in all the cases, which suggested the involvement of the mechanisms destroying the tumor tissue due to hyperthermia and PDT effects. The only exclusion was focus 3, for which the irradiation mode was initially incorrect and the dose delivered in 8 min was less than required. The non-invasive temperature monitoring was a good indicator of the necessity to correct the mode. Once the adjustment was done, the dose was fully delivered.

Thus, both the dual-wavelength fluorescence and noninvasive contact-free temperature monitoring may be considered to be effective tools of PDT procedure control while the results of the PDT monitoring to be predictors of treatment outcome.

### Remote treatment results.

The combined usage of dual-wavelength fluorescence monitoring and noninvasive contactless thermometry in the course of photodynamic therapy of BCSC allows for simultaneous control of the photosensitizer photobleaching level and the depth of the photodynamic action on the tissue as well as the degree of the involvement of the mechanisms, associated with hyperthermia, as well as the correctness of the procedure performance. Application of bimodal monitoring provides the opportunity to adjust the irradiation mode in the course of the procedure in order to achieve optimal results and to make the procedure more comfortable for a patient.

**Figure 7:**
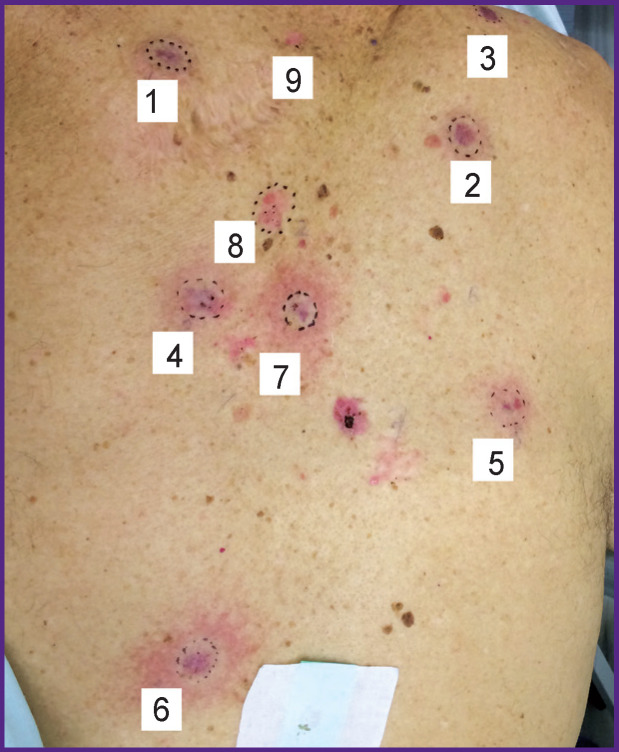
Foci of basal cell skin cancer immediately after the session of the photodynamic therapy

Clinically, after the PDT procedure, erythema as the manifestation of local hyperthermia was noted. The cyanotic color indirectly indicated blood supply impairment in the affected skin areas due to the development of the intravascular erythrocyte aggregation (Figure 7).

**Figure 8:**
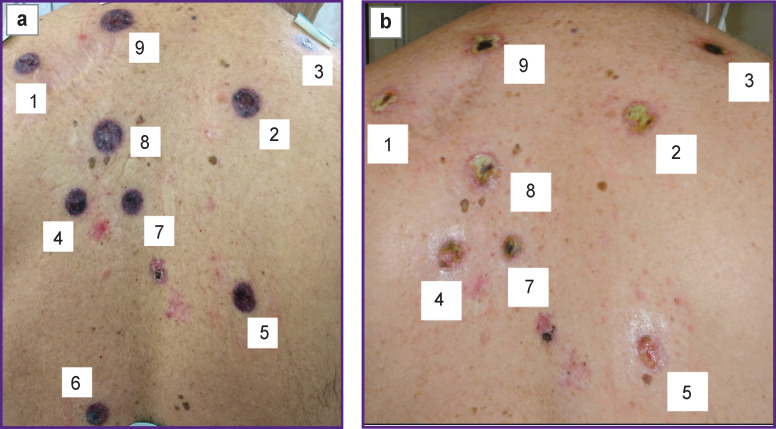
Foci state after the photodynamic therapy of basal cell skin cancer:

During dynamic follow-up, colliquative necrosis with sharp boundaries developed predictably in all BCSC foci 1 week after the PDT procedure (Figure 8 (a)). A month after the PDT procedure, ulcerative defects with rejection of the necrotic masses and initial stages of epithelization were predictably observed in all irradiated areas (Figure 8 (b)).

(а) development of tissue necrosis 7 days after the procedure; (b) epithelization of the defects 1 month after the procedure

**Figure 9:**
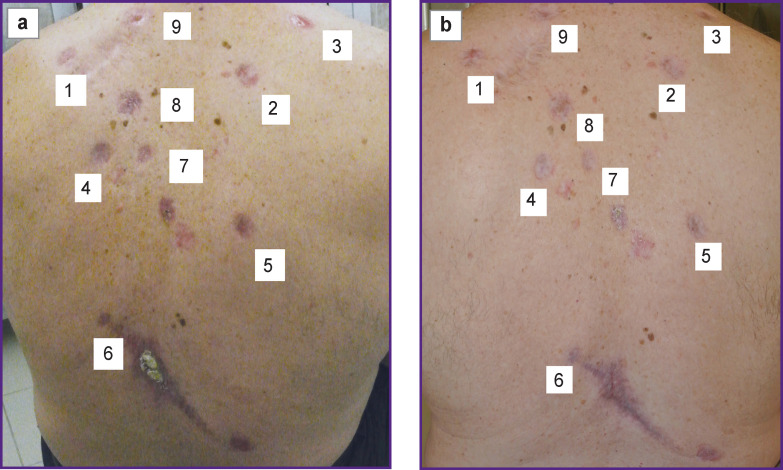
Scar alterations in the foci after the photodynamic therapy of basal cell skin cancer: (а) 4 months later; (b) 6 months later

Four months after the PDT session, all 9 treated skin defects were healing by secondary intention with formation of connective tissue scars (Figure 9 (a)). In half a year after the PDT procedure, normotrophic scars were formed in the places of BCSC foci, clinical and dermatoscopical signs of local recurrence were absent (Figure 9 (b)).

## Conclusion

A new approach to non-invasive monitoring of the PDT procedure efficacy using chlorin-based photosensitizers has been proposed consisting in the combined application of dual-wavelength fluorescence imaging and non-invasive temperature control. This approach was clinically tested on a patient treated for multiple BCSC foci. The dual-wavelength fluorescence monitoring has been shown to control both the photosensitizer accumulation and bleaching, and at the same time, the complementary application of two excitation wavelengths makes it possible to evaluate the depth of photosensitizer accumulation and procedure performance, whereas the contactless temperature monitoring allows one to control the correctness of the conducting procedure and temperature mode. On the basis of the information obtained it is possible to predict the results of the therapy and adjust intraoperatively the parameters of laser impact in order to achieve positive results taking into account individual reactions of each patient.
